# Fidelity, adaptation and integration of whole-school health promotion within Dutch schools: a cross-sectional survey study

**DOI:** 10.1093/heapro/daad173

**Published:** 2023-12-20

**Authors:** Gerjanne Vennegoor, Patricia van Assema, Gerard R M Molleman, Pepijn van Empelen, Joyce Dieleman, Maria W J Jansen

**Affiliations:** Academic Collaborative Center for Public Health Limburg, P.O. Box 33, 6400 AA Heerlen, The Netherlands; Department of Health Promotion, Research Institute of Nutrition and Translational Research in Metabolism (NUTRIM), Maastricht University, P.O. Box 616, 6200 MD Maastricht, The Netherlands; Academic Collaborative Center for Public Health Limburg, P.O. Box 33, 6400 AA Heerlen, The Netherlands; Department of Health Promotion, Research Institute of Nutrition and Translational Research in Metabolism (NUTRIM), Maastricht University, P.O. Box 616, 6200 MD Maastricht, The Netherlands; Department of Primary and Community Care, Academic Collaborative Center AMPHI, Radboud Institute for Health Sciences, Radboud University Medical Center, P.O. Box 9101, 6500 HB Nijmegen, The Netherlands; Department of Healthy Living, Public Health Service Gelderland-Zuid, P.O. Box 1120, 6501 BC Nijmegen, The Netherlands; Expertise Center Child Health, Netherlands Organization for Applied Scientific Research (TNO), P.O. Box 3005, 2301 DA Leiden, The Netherlands; Public Health Service Noord- en Oost Gelderland, Academic Collaborative Center AGORA, P.O. Box 3, 7200 AA Zutphen, The Netherlands; Academic Collaborative Center for Public Health Limburg, P.O. Box 33, 6400 AA Heerlen, The Netherlands; Department of Health Services Research, Care and Public Health Research Institute (CAPHRI), Maastricht University, P.O. Box 616, 6200 MD Maastricht, The Netherlands

**Keywords:** health promoting school, implementation, HPS implementation questionnaire, the Netherlands, survey study

## Abstract

Implementing comprehensive health promotion programs in the school setting can be challenging, as schools can be considered complex adaptive systems. As a first step towards understanding what works in improving implementation for which schools and under which conditions, this study aimed to examine the degree of implementation of health promoting school (HPS) programs, in terms of five dimensions of fidelity (*adherence, dose, participant responsiveness, quality of delivery* and *program differentiation*), and the dimensions of *adaptation* and *integration*. The HPS Implementation Questionnaire was distributed among ± 2400 primary, secondary, secondary vocational and special needs schools in the Netherlands. Employees of 535 schools (22.3%) filled out the questionnaire. Data were analysed by descriptive statistics and ANOVA tests. The average degree of implementation was 2.55 (SD = 0.58, range = 0.68–3.90; scaled 0–4). The lowest scores were achieved for *participant responsiveness* and *adherence,* and the highest for *integration* and *adaptation*. Schools that identified as HPS reported significantly higher overall degree of implementation, *adherence*, *dose*, *participant responsiveness*, *program differentiation* and *adaptation* than schools that didn’t. Primary schools achieved a significantly higher degree of implementation, *dose*, *participant responsiveness*, *quality of delivery* and *integration* than other school types. In conclusion, many schools work on student health and well-being to some extent, but the vast majority have much room for improvement. Higher implementation scores for schools that identified as HPS underline the value of HPS programs. A broader perspective on health and more insight into conditions for effectiveness and implementation in secondary and secondary vocational schools are needed.

**Contribution to Health Promotion**

- Implementation of comprehensive health promoting programs in schools can be challenging, due to the dynamic practice in and around schools.
- Using a survey, we showed that many schools include health promotion in their routines, and often apply a flexible approach.
- Fewer schools managed to actively involve everyone, and implement health promotion in all four core areas: education, school environment, referral and policy.
- Schools that identified as ‘health promoting school’ performed better. This confirms that such programs help schools with the implementation.
- Primary schools performed better than secondary and secondary vocational schools. This calls for more research to understand why.

## BACKGROUND

Schools are a key setting for promoting health behaviors, health and well-being of students during their school career, potentially even leading to improved academic achievements (WHO, 1997; Suhrcke and de Paz Nieves, 2011). As such, schools can play an important role in affecting lifestyle and health later in life (Conti, Heckman, and Urzua, 2010). Moreover, apart from students, other target groups can be affected in the school setting, such as staff and families (Gugglberger, 2021). Therefore, around 35 years ago, the health promoting school (HPS) framework was developed, consisting of a whole-school approach including healthy school policies, health education in the curriculum, a healthy physical and social environment, engagement with the community and health services (WHO, 1986, 2021b; Langford et al., 2014; Lewallen et al., 2015). The comprehensive framework demands system changes that are integrated (or ‘added in’) into the school rather than ‘added on’ to core educational tasks (Inchley, Muldoon, and Currie, 2007; Mcnab, 2014; Bentsen et al., 2020; Gugglberger, 2021). Programs grounded in the HPS framework show promising evidence in improving health behaviors and health in students around the world (e.g. body mass, physical activity and bullying), but the effects remain inconsistent (Langford et al., 2014; Turunen et al., 2017). This may be due to varying and limited degrees of implementation (Langford et al., 2014; Turunen et al., 2017; Darlington, Violon, and Jourdan, 2018). Nevertheless, there is broad recognition for increasing the adoption and sustainability of HPS programs globally (Herlitz et al., 2020).

While implementation of all health promotion programs can be challenging, it especially applies to programs seeking a whole-school system change as for HPS (Samdal and Rowling, 2011). This is because the school can be seen as a complex adaptive system, in which there is constant interaction between its many components (e.g. student wellbeing, staff turnover and municipal policy) (e.g. Keshavarz et al., 2010; Rosas, 2015; Bartelink, 2019). That forms a strongly dynamic, non-linear context, resulting in a continuously changing system that is unique to each school. Therefore, each school responds to (health promotion) changes in its own way, thereby challenging the implementation of whole-school HPS programs (Nutbeam, 1992; Durlak and DuPre, 2008; Turunen et al., 2017; Darlington, Violon, and Jourdan 2018; Gugglberger, 2021; WHO, 2021a, 2021b). Consequently, the implementation process should not only strive for fidelity [i.e. implementation as intended by program developers (Dusenbury et al., 2003)] but also allow room for adaptation of ‘peripheral’ elements to the school context as well as integration (‘adding in’) into the school system (Durlak and DuPre, 2008; Kremser, 2010; Patton, 2011; Baumann, Cabassa, and Stirman, 2017; Darlington, Violon, and Jourdan, 2018; Bartelink and Bessems, 2019; Gugglberger, 2021).

Research into the degree to which whole-school HPS implementation, in terms of fidelity, adaptation and integration, is achieved has been limited in terms of quantity as well as perspective (Samdal and Rowling, 2011; Langford et al., 2014; Turunen et al., 2017; Gugglberger, 2021). Studies generally use a narrow framework focusing only on the first two out of five dimensions of fidelity: (i) *adherence* to the HPS framework, (ii) *dose* or intensity received by participants, (iii) *participant responsiveness*, i.e. engagement of participants, (iv) *quality of delivery* of the program and (v) *program differentiation*, i.e. the degree of uniqueness of the program (Dusenbury et al., 2003; Langford et al., 2014). Adaptation to the specific circumstances and characteristics of the school context (Baumann, Cabassa, and Stirman, 2017) and integration, i.e. the extent to which the approach is part of school routines, norms and identity (Gugglberger, 2021), are rarely (thoroughly) assessed (Langford et al., 2016; Lambert et al., 2017; Schaap et al., 2018; Toomey et al., 2020; Vennegoor et al., 2022). Moreover, while there seems to be a step-wise increase in percentages of schools identifying as HPS for secondary vocational, secondary and primary education, it is unclear if and how implementation levels differ between these school types (Vilaca et al., 2019; Bartelink, Bessems, and Prevo, 2020). Furthermore, in Europe, a study among a sample of national HPS coordinators showed that 82% of the included countries have national or regional institutional tools or resources to support HPS implementation, 63% have incorporated HPS in three or more national policies and 42% have some form of national certification (Vilaca et al., 2019). Yet, there are many unknowns, for instance regarding how many schools identified as HPS and which topics are addressed (Vilaca et al., 2019; Gugglberger, 2021).

In the Netherlands, the HPS framework is reflected in the Healthy School Program (‘Programma Gezonde School’; HS Program). The national program supports schools in implementing a whole-school approach consistent with the framework (van Koperen et al., 2020). Since 2019, a 4-year evaluation study has been conducted into conditions for the effectiveness of the HS Program (Vennegoor et al., 2020). As part of this evaluation, a comprehensive instrument to measure the degree of implementation, in terms of fidelity, adaptation and integration, was developed in an earlier phase of the study (Vennegoor et al., 2022). Consequently, the present study aimed to: (i) get insight into the actual degree of implementation of school health promotion, according to seven dimensions, at Dutch primary, secondary, secondary vocational and special needs schools in the 2019/2020 school year, and (ii) investigate whether schools which identified as (certified) HPS show a higher degree of implementation.

## METHODS

### Dutch education system and school health promotion

In the Netherlands, children generally go to primary school at the age of 4. After 8 years, students proceed to one of four types of secondary education: practical education (4–6 years), pre-vocational secondary education (4 years), senior general secondary education (5 years) or pre-university education (6 years). Most pre-vocational secondary students, and some practical and senior general secondary education students, continue to secondary vocational education (1–4 years). Students with learning difficulties can go to one of three types of special needs schools: special schools for primary or secondary education (at which students receive more guidance during the learning process), or schools for special education (for students with a disability, chronic illness or serious behavioral problems). There are limited legal requirements for schools regarding health promotion, e.g. a protocol to prevent bullying and smoke-free school grounds.

The national HS Program is funded by the government (ministries of health, education, social affairs and agriculture) and coordinated by five national organizations in the education and public health sector. Within each of the Public Health Service (PHS) regions in the country, ‘HS advisers’ recruit schools and offer support. Schools are encouraged to implement a whole-school approach by focusing on four so-called pillars: (i) education to promote knowledge and skills, (ii) a healthy physical and social school environment, (iii) identification of students who need extra attention or referral and (iv) health promotion as part of school policy. They choose one or more of the 10 topics to implement. Further details of the HS Program were described elsewhere (van Koperen et al., 2020; Vennegoor et al., 2022).

Schools can apply for a 3-year topic certificate if they adhere to all corresponding quality criteria. With their first topic certificate, they also attained the general HS Program certificate [19% of schools in 2019 (Programma Gezonde School, 2020)]. Certificates are not mandatory and only available for five topics, and HS advisers indicate there is an (unknown) higher percentage of schools that implemented the HS Program. Regardless of HS certification status, other whole-school approaches and evidence-based interventions are implemented in Dutch schools (e.g. Willeboordse et al., 2016; Busch et al., 2018; van Koperen et al., 2020).

### Study setting and design

Invitations for the cross-sectional online survey study were aimed at all primary, secondary, secondary vocational and special needs schools in the seven (out of 25) participating PHS regions in the evaluation study (approximately 2400, or 27%, out of 9000 schools in the country). Regions were selected based on variation in geographical location and willingness to participate (Vennegoor et al., 2020). The targeted sample was one employee per school, who was most knowledgeable about the school’s approach to student health and well-being (according to the respondent’s own judgment; Vennegoor et al., 2022). Ethical clearance was provided by the Ethics Review Committee of the Faculty of Health, Medicine, and Life Sciences of Maastricht University.

### Recruitment

Schools were recruited from September through November 2020. Recruitment was performed in close collaboration with HS advisers and preventive youth health physicians of the PHSs. The process was pre-discussed during a meeting of the evaluation study’s Community of Practice (CoP; *N* = 12 practice professionals), in which researchers and practice professionals (such as HS advisers of PHSs and employees of national organizations in the health and education sector) exchange information as part of a mutual learning process (Wenger, 1998; Vennegoor et al., 2020). As PHS contexts vary, CoP members preferred for each PHS to write their own recruitment plan. Final plans included sending a (personalized) email (seven regions) and reminder (three) to all schools, adding the invitation to a newsletter (six), handing out postcards during regular school visits or inviting schools during regular phone calls (five), sending postcards to all schools (two) and/or posting on social media (two). Instructions, draft messages, postcards and biweekly response updates were provided by the research team. After 2 months, the team evaluated response rates with every PHS, and, if needed, subsequent changes in focus were made (e.g. additional recruitment among special needs schools or schools without an HS Program certificate). To compensate for their efforts, every PHS received a financial contribution as well as a report on descriptive statistics on the seven dimensions for schools in their region. All results were also presented during two meetings of the evaluation study’s CoP, following joint (sub)group interpretation of the main results.

Additionally, schools were recruited via the website, newsletter and/or social media messages of three national education councils, four knowledge institutes (e.g. on nutrition or employee working conditions), two labor unions, two academic collaborative centers and the HS Program, as well as via the network of the research team.

## MEASURES

### HPS implementation questionnaire

The degree of implementation was assessed with the HPS implementation questionnaire, which was developed in close collaboration with CoP experts in the field of school health promotion in the Netherlands in an earlier phase of the evaluation study (Vennegoor et al., 2022). It was shown to be internally consistent with α’s > 0.72 for subscales and α = 0.90 for the full scale. The questionnaire can be used to differentiate in the degree of HPS implementation, in terms of fidelity, adaptation and integration, between schools or within one school over time. The absolute scores are less informative, but they serve to identify areas of improvement within the dimensions. Additionally, they can provide a ranking based on the scores obtained by the sample of schools or within one school at various time points.

Supplementary File 1 provides an overview of the full questionnaire. The 28 items cover seven implementation dimensions (adherence, dose, participant responsiveness, quality of delivery, program differentiation, adaptation and integration), each measured by 1–12 items (Dusenbury et al., 2003; Baumann, Cabassa, and Stirman, 2017; Vennegoor et al., 2022). Confirmatory factor analysis previously showed that the items can best be reflected by the seven subscale scores (one for each dimension; Vennegoor et al., 2022). Scores are presented on a scale from 0 to 4, and a 4 can be considered an optimal score. A weighted average of these scores results in one ‘degree of implementation’ score, which can be used to get an additional indication of the overall implementation [based on psychometric analysis, program differentiation and adaptation are assigned half the weight of the other subscales (Vennegoor et al., 2022)]. Additionally, examining each item separately can facilitate a deeper understanding of subscale results. Within subscale *adherence*, there are eight topic scores, reflecting integral adherence for a topic according to the pillars of the HS Program (see Supplementary File 1).

At the beginning of the questionnaire, the following background items were added: school name (open-ended), municipality (multiple choice), school type (multiple choice), professional role (multiple choice), high knowledgeability on the school approach towards health and wellbeing (yes/ no) and identification as HPS (yes/no/don’t know) (see Supplementary File 1 for the complete questionnaire).

The questionnaire was entered into Qualtrics software (Qualtrics, 2020). The estimated response time was 10–15 min (Vennegoor et al., 2022). Questions were answered concerning the 2019/2020 academic year before the COVID lockdown (August 2019 until February 2020). After completion, respondents could indicate whether they would like to receive a report with their scores in each of the seven dimensions relative to all participating schools.

### Background information

Additional information at the school level was collected from several existing databases. A certification database of the HS Program, containing all granted topic and program certificates from July 2011 until September 2020, was provided by the program. Open access data on the level of urbanicity (low: < 1000 addresses per km²; medium; high: > 1500) in January 2020 for every zip code in the Netherlands were downloaded from Statistics Netherlands (CBS) (Statistics Netherlands, 2020; van Leeuwen, 2020). Open access data on the number of students and school denominations (public, independent non-denominational, Catholic, protestant, collaboration or other) in October 2020, and the percentage of primary school students with two lower educated parents in the 2018/2019 academic year, were derived from governmental organization *Dienst Uitvoering Onderwijs* (DUO) (DUO, 2020).

### Data processing and statistical analysis

Data were analysed using SPSS software (V.27.0; Armonk, NY, USA). Background data were merged with questionnaire data based on manually added school codes from DUO open access data (DUO, 2020). If duplicate responses for one school were received, because of multiple employees responding or one employee responding twice, a protocol was followed to select one response (e.g. based on completeness).

As respondents could select multiple school types, this was recoded as follows: primary (if primary school and/or special school for primary education), secondary (if secondary school, or combined with primary school), secondary vocational (if secondary vocational school, or combined with secondary school) and special needs (if special school for secondary education and/or school for special education, or combined with any other types). HS categories were coded as: No HS (schools that didn’t identify as HPS at all), Partial HS (schools that identified as HPS according to their own response—see Supplementary File 1, Q7) and Certified HS (schools whichever obtained a program certificate).

Descriptive analyses were used to describe background information. In order to map the degree of implementation of school health promotion, in terms of the seven dimensions, among all participating schools, descriptive analyses were used on all item and subscale scores of the HPS implementation questionnaire. As no cut-off values exist due to the relative assessment, results were described by indicating the lowest and highest average scores among the items within one subscale. All item and topic scores were rescaled to a 0–4 range to facilitate interpretation (see Supplementary File 1 for the scoring scheme). Subscale scores and degree of implementation were only calculated for complete (sub)scale responses (including don’t know answers). Don’t know answers (only possible for 16 out of 28 items) were coded as missing data for the descriptive analyses of items to reflect the most accurate results. These answers were all imputed with zero when calculating subscale scores as it concerned only a few schools (generally < 0.05%), this response was most accurate based on the other responses for over 90% of these schools, and there was roughly equal division over the school types and HS categories. To examine differences between the four school types as well as between HS categories, interaction between these variables was tested first. As two-way (education * HS category) ANOVA tests showed no significant interaction (*p*’s > 0.03; α = 0.01 to account for multiple testing), one-way ANOVA tests were used (or Welch’s ANOVA if Levene’s test was significant at α = 0.05). Differences between the overall degree of implementation were then tested for as a first indication. Subsequently, the main ANOVA analyses were performed on the seven subscale scores. To provide additional insights into variation between categories, descriptive analyses were performed on all items.

With regard to the ANOVA tests, a *p*-value < 0.01 (to account for multiple testing) was considered significant and was followed up with post-hoc testing. School types were then compared by Hochberg tests (or Games-Howell if Welch’s), and HS categories by planned contrasts (contrast 1: Certified and Partial HS vs. No HS; contrast 2: Certified HS vs. Partial HS). Effect sizes were calculated for ANOVA (ω²), Hochberg ($g_{hedges}$) and planned contrasts (*r*) and categorized into small (ω² = 0.01; $g_{hedges}$ = 0.2; *r* = 0.1), medium (ω² = 0.06; $g_{hedges}$ = 0.5; *r* = 0.3) or large effect size (ω² = 0.14; $g_{hedges}$ = 0.8; *r* = 0.5) (Cohen, 1988; Kirk, 1996).

## RESULTS

### Sample

Employees of 535 schools (22.3% of invited schools) filled out the questionnaire, of whom 418 (78.1%) provided a complete response (see Supplementary File 2). Out of all complete responses, 363 (86.8%) elected to receive a report. Duplicate responses were received for 79 schools, of which 56 were removed based on completeness and knowledgeability on the topic, and 23 based on later responses in time (to limit recall bias).

Schools were divided into 365 primary (21.5% of those invited), 102 secondary (28.2%), 25 secondary vocational (13.2%) and 43 special needs schools (27.9%). In total, 30.5% were categorized as No HS, 26.9% as Partial HS and 42.6% as Certified HS. The distribution of school types in Partial and Certified HS groups was comparable, but the No HS group consisted of more primary (76.7% vs. 60.4% and 58.3%) and fewer secondary schools (9.8% vs. 23.6% and 22.8%). Most respondents selected the role of the school principal (38.5%; mainly at primary schools), teacher/lecturer (18.9%; mainly at secondary schools), HS coordinator (18.9%; mainly at secondary vocational and special needs schools) and/or school counselor (12.7%). Additional characteristics show most schools were Catholic (42.5%) and were situated in an area of low urbanicity (45.9%; Supplementary File 2).

### Degree of implementation of whole-school health promotion

Supplementary File 3 (Table 2, column ‘Total’) details the average (sub)scale and item scores of all schools (all scaled 0–4). A first indication of the degree of implementation can be obtained from the overall score. The average degree of implementation score was 2.55, ranging between 0.68 and 3.90 (SD 0.58).

The main indicators for the degree of implementation are the seven subscale scores reflecting fidelity, adaptation and integration. On average, the highest scores were achieved for subscales *integration* (2.95) and *adaptation* (2.82). Subscales *quality of delivery* (2.66), *dose* (2.65) and *program differentiation* (2.62) were the middle scores. The lowest average scores were obtained for *participant responsiveness* (2.44) and *adherence* (2.00). Schools obtained scores on nearly the full range of each subscale.

Additional insights can be obtained from the variation in item scores. The eight topic scores within subscale *adherence*, measuring the delivery of program components in accordance with the HPS framework, revealed that topics well-being (2.71) and physical activity (2.00) were best, and environment (0.83) and relations and sexuality (1.09) were least adhered to. Detailed adherence scores in Supplementary File 4, Table 3, show that for topics of well-being and prevention of hearing damage, the lowest proportion of schools use educational activities. For all other topics, a particularly low proportion of schools conduct a recurrent measurement of student health on the topic. The five final *adherence* items show the highest average scores for appointing one or more coordinators for the school approach towards health and wellbeing (2.97). Annual evaluation and availability of sufficient hours and budget for those responsible received the lowest scores (1.49, 2.28 and 2.32 on average).

Regarding subscale *dose,* assessing the intensity as received by participants, schools scored highest on employees complying with the behavioral rules as well as being a good example for students (3.04 and 2.90 on average). The lowest average scores were obtained for having regular agenda items and active communication with employees (2.20 and 2.39). In subscale *participant responsiveness,* evaluating engagement of participants, school principals (3.12) and coordinators of the school’s approach towards health and wellbeing (3.06) were on average most actively involved. Management (1.83), parents (2.01) and external advisers (2.05) were least actively involved. Regarding subscale *quality of delivery*, the expertise of external professionals and teachers was on average considered the highest (3.14 and 2.91). Contact with external supporter(s) as well as onboarding of new employees were considered the lowest (2.31 and 2.36 on average). Compared to other subscales, *integration* shows high scores on all items (≥2.72). Items display that, on average, respondents obtained the highest scores for the school approach being in line with the school vision (3.21).

### Differences between school types

The first ANOVA test on differences between school types revealed a medium-size effect of school type on the overall degree of implementation score (*F*(3, 414) = 10.134, *p* < 0.001, ω² = 0.06). On average, primary schools showed a higher degree of implementation of whole-school health promotion than secondary and secondary vocational schools (Table 1).

**Table 1:** Differences in implementation dimensions between school types

Dimension	Total		Primary schools		Secondary schools		Secondary vocational schools		Special needs schools		F	Post-hoc
	*N*	*M (±SD)*	*N*	*M (±SD)*	*N*	*M (±SD)*	*N*	*M (±SD)*	*N*	*M (±SD)*		
Adherence	429	2.00 (±0.58)	298	2.01 (±0.60)	76	1.98 (±0.53)	22	1.86 (±0.51)	33	2.05 (±0.53)	0.580	-
Dose	438	2.65 (±0.75)	302	2.84 (±0.69)	80	2.19 (±0.75)	22	1.97 (±0.49)	34	2.51 (±0.61)	26.902**	PS > **SS**, **SVS**, SNS SNS > **SVS**
Participant responsiveness	433	2.44 (±0.71)	300	2.53 (±0.72)	77	2.27 (±0.68)	22	2.16 (±0.53)	34	2.27 (±0.59)	5.042*	PS > SS
Quality of delivery	418	2.66 (±0.72)	291	2.77 (±0.72)	72	2.41 (±0.64)	22	2.18 (±0.63)	33	2.48 (±0.68)	10.080**	PS > SS PS > SVS
Program differentiation	418	2.62 (±1.00)	288	2.66 (±1.02)	75	2.63 (±1.08)	22	2.18 (±0.80)	33	2.58 (±0.79)	2.335^W^	-
Adaptation	418	2.82 (±0.93)	288	2.88 (±0.92)	75	2.69 (±0.99)	22	2.41 (±0.91)	33	2.79 (±0.86)	2.353	-
Integration	418	2.95 (±0.66)	288	3.04 (±0.65)	75	2.83 (±0.62)	22	2.48 (±0.73)	33	2.71 (±0.53)	8.180**	PS > SVS PS > SNS
Degree of implementation	418	2.55 (±0.58)	288	2.65 (±0.57)	75	2.36 (±0.57)	22	2.13 (±0.50)	33	2.43 (±0.48)	10.134**	PS > SS PS > SVS

Scale: 0–4. Effect size ANOVA/Welch: ω². Post-hoc: Hochberg/Games-Howell, using $g_{hedges}$ as effect size. ^W^ = Welch statistic, PS = primary schools, SS = secondary schools, SVS = secondary vocational schools, SNS = special needs schools, *M* = mean, SD = standard deviation, bold = medium or large effect size, ** = *p* < 0.001, * = *p* < 0.01.

Main ANOVA tests on the seven subscales showed a large effect of school type on subscale *dose* (*F*(3, 434) = 26.902, *p* < 0.001, ω² = 0.15). Among primary schools, participants received a significantly higher intensity than all other school types, and special needs schools reached a significantly higher intensity compared to secondary vocational schools (Table 1). Further descriptive analysis of item scores shows school types varied in particular in active communication to employees, ranging between 1.36 on average for secondary vocational and 2.61 for primary schools (Supplementary File 3, column ‘School type’).

A medium-size effect was found for subscale *quality of delivery* (*F*(3, 414) = 10.080, *p* < 0.001, ω² = 0.06), with primary schools achieving significantly higher quality than secondary schools and secondary vocational schools. The extent to which new employees were familiarized with the school’s approach varied most (1.45 on average at secondary vocational and 2.63 at primary schools).

Subscales *participant responsiveness* and *integration* revealed small significant effects between school types (Table 1). Primary schools had significantly higher participant engagement than secondary and secondary vocational schools. Item scores show involvement of parents varied the most (1.00 at secondary vocational and 2.20 at primary schools), whereas there was the least variation in student involvement (2.09 and 2.40). Primary schools also had a significantly higher level of integration than secondary vocational and special needs schools. On all those items, primary schools show the highest and secondary vocational schools the lowest average scores as well, with the largest variation in the item on alignment with the school’s vision (3.30 and 2.55).

There were no significant differences between school types for subscales *adherence, adaptation* and *program differentiation*. Descriptive analysis did show a large variation in *adherence* topic scores and particularly for *smoking, alcohol & drugs*, ranging between 0.67 on average for primary and 2.65 for secondary vocational schools.

### Differences between HS categories

When comparing HS categories (No HS, Partial HS and Certified HS), a small subgroup effect was found for the overall degree of implementation (*W*(2, 231) = 12.235, *p* < 0.001, ω² = 0.06). Certified and Partial HS achieved a significantly higher degree of implementation than No HS, and Certified HS a significantly higher degree than Partial HS (Table 2).

**Table 2:** Differences in implementation dimensions between HS categories

Dimension	Total		No HS		Partial HS		Certified HS		F	Contrast^a^
	*N*	*M (±SD)*	*N*	*M (±SD)*	*N*	*M (±SD)*	*N*	*M (±SD)*		
Adherence	429	2.00 (±0.58)	130	1.78 (±0.64)	105	1.99 (±0.51)	194	2.16 (±0.53)	**15.590** ^W^**	1 + 2
Dose	438	2.65 (±0.75)	134	2.48 (±0.82)	105	2.60 (±0.67)	199	2.80 (±0.71)	7.718**	1 + 2
Participant responsiveness	433	2.44 (±0.71)	133	2.21 (±0.80)	105	2.41 (±0.66)	195	2.62 (±0.62)	13.498 ^W^**	1 + 2
Quality of delivery	418	2.66 (±0.72)	120	2.53 (±0.78)	104	2.60 (±0.68)	194	2.77 (±0.68)	4.65	-
Program differentiation	418	2.62 (±1.00)	123	2.33 (±1.09)	101	2.65 (±0.88)	194	2.79 (±0.97)	8.072**	1
Adaptation	418	2.82 (±0.93)	123	2.59 (±1.09)	101	2.78 (±0.78)	194	2.97 (±0.86)	5.750^W^*	1
Integration	418	2.95 (±0.66)	123	2.82 (±0.70)	101	2.92 (±0.60)	194	3.04 (±0.65)	4.24	-
Degree of implementation	418	2.55 (±0.58)	123	2.36 (±0.66)	101	2.51 (±0.51)	194	2.69 (±0.53)	12.235^W^**	1 + 2

Scale: 0–4. Effect size ANOVA/Welch: ω². Post-hoc: planned contrasts, using *r* as effect size.

^a^Contrast 1: Certified and Partial HS vs. No HS; contrast 2: Certified HS vs. Partial HS. ^W^ = Welch statistic, HS = Healthy School, *M* = mean, SD = standard deviation, bold = medium or large effect size, ** = *p* < 0.001, * = *p* < 0.01.

Main analyses revealed a medium effect on subscale *adherence* (*W*(2, 244) = 15.590, *p* < 0.001, ω² = 0.07). Planned contrast comparisons showed significantly higher adherence to the HPS framework for Certified and Partial HS than for No HS, as well as higher adherence for Certified HS than Partial HS (Table 2). Descriptive analysis of topic scores revealed the most variation for topics nutrition (1.38 for No HS and 2.26 for Certified HS on average) and physical activity (1.66 and 2.28; Supplementary File 3, column ‘HS category’). Variation was shown on all pillars, but the largest differences are in the proportion of schools conducting recurrent measurement of student health, which is about three times higher for Certified HS than No HS (24 and 6% HS for nutrition; 42 and 15% for physical activity). Among certified HS schools, higher topic scores were reached if the corresponding topic certificate was obtained rather than other certificate(s), with the smallest difference for well-being and the largest for smoking, alcohol, and drugs (Supplementary File 4, Table 4). On the five other adherence items, the largest variation was observed in having one or more HS coordinators appointed (2.28 for No HS and 3.44 for Certified HS on average).

Four subscales revealed small effects, with Certified and Partial HS achieving significantly higher *dose*, *participant responsiveness*, *program differentiation* and *adaptation* than No HS (Table 2). Certified HS also had significantly higher *dose* and *participant responsiveness* than Partial HS. Within items on intensity, subgroups varied in particular in the frequency of agenda items for employees (1.88 for No HS and 2.45 for Certified HS) and reaching (almost) all students (2.49 and 2.98). Regarding the engagement of participants, most variation was found in the average level of involvement of an external adviser (1.42 and 2.51) and the HS coordinator (2.56 and 3.48).

There were no significant differences between HS categories for subscales *quality of delivery* and *integration*. Certified HS scored highest on all items, and No HS lowest on most quality and all integration items.

## DISCUSSION

The present study aimed to examine the implementation of whole-school health promotion at Dutch primary, secondary, secondary vocational and special needs schools that do and don’t identify as (certified) HPS. Results show large variation between schools on the overall degree of implementation as well as on most items and all seven dimensions that were assessed (*adherence*, *dose*, *participant responsiveness*, *quality of delivery*, *program differentiation*, *adaptation* and *integration*). Although some schools hardly work on student health and well-being, most schools did implement school health promotion to some extent. Nonetheless, only a small group achieved (near) optimal implementation and the vast majority has much room for improvement.

Of the seven dimensions, schools are already working most on *adaptation* of the whole-school approach to the specific circumstances and characteristics of the school context and *integration*, i.e. the extent to which the approach is part of school routines, norms and identity. Within the other five dimensions, aspects that seem to be succeeding more are related to effective internal coordination as well as competences of employees (e.g. giving a good example). Regarding the eight health promotion topics assessed, well-being was best adhered to. According to CoP members and similar to a study into perceptions of HPS (Keshavarz Mohammadi, Rowling, and Nutbeam, 2010), these results may mainly be explained by the close link between student wellbeing and the pedagogical vision of all schools, making it easier to incorporate these (aspects of) dimensions.

Overall, the dimension that was least achieved was *adherence* to the HPS framework. Adherence varied between topics and was particularly low for topics environment and relations and sexuality. For these and most other topics, the main limitations were in recurrent measurement of student health as well as available resources, as was seen in previous studies (e.g. Belansky et al., 2013; Joyce et al., 2017). Moreover, schools seem to focus on urgent topics for their student population (e.g. drug use at secondary schools), rather than having a broader perspective from the start. In the other six dimensions, aspects related to continuous engagement of employees (e.g. in terms of regular agenda items) and professional support showed much room for improvement. Future HPS implementation research and practice may, therefore, provide more guidance on how to achieve such broader implementation involving all actors in the complex school system as well as how to increase monitoring and resources (Deschesnes, Martin, and Hill, 2003; Adamowitsch, Gugglberger, and Dür, 2014; Joyce et al., 2017). The findings on differences across the seven dimension scores might apply to other programs and settings as well, for instance worksite health promotion programs (Wierenga et al., 2013), but future research applying a comprehensive view is needed to examine these potential similarities.

There were differences between schools with regard to identification as HPS (No, Partial, Certified). Those which identified as HPS had a higher degree of implementation, *adherence, dose, participant responsiveness, program differentiation* and *adaptation*. In line with previous research, though, certified schools had higher scores than non-certified schools (i.e. Certified > Partial HPS) for fewer dimensions and only for those which certification criteria focus on (Keshavarz Mohammadi, Rowling, and Nutbeam, 2010; Joyce et al., 2017; Verjans-Janssen et al., 2020). Aspects related to the roles of an (HS) coordinator and an external adviser were most distinctive between the three categories, and the importance of these roles has been shown before (Boot, 2010; Gleddie, 2011; Adamowitsch, Gugglberger, and Dür, 2014; Verjans-Janssen et al., 2020; Bartelink et al., 2022). Moreover, regarding *adherence*, the largest differences were shown for three topics: nutrition, physical activity and smoking, alcohol and drugs. Except for well-being, which seems to be a priority in all schools, schools that identified as HPS often focus less on other topics, such as environment and media literacy (Langford et al., 2014; Bartelink, Bessems, and Prevo, 2020; Programma Gezonde School, 2020). Considering the importance of all topics, there is still room for improvement toward a broader perspective on health in the implementation of HPS programs.

The distinction between the three categories was modest, which may be because the assessed dimensions are not unique to the HS Program, and many schools likely (also) implemented other (whole-school) health promotion programs (e.g. Willeboordse et al., 2016; Busch et al., 2018; van Koperen et al., 2020). However, the consistent pattern of higher scores for schools that identify as (certified) HPS does support the value of HPS programs in assisting to work towards broad implementation. Nevertheless, there were still some ‘Partial’ and ‘Certified’ schools with lower scores as well as a group of ‘No HS’ with (near) optimal scores. This indicates that identification as HPS is only one of the potential conditions for (optimal) implementation. Other conditions may be more important in some schools and/or at certain times. Future research is essential to understand what works for which schools and under which conditions, in order to find ways to further strengthen the implementation process and reduce heterogeneity in effects (Gleddie, 2011; Langford et al., 2015; Paulussen, 2017; Darlington, Violon, and Jourdan, 2018; Vennegoor et al., 2020).

Finally, several clear differences were shown between primary, secondary, secondary vocational and special needs schools. Primary schools achieved a higher average degree of implementation, *dose, participant responsiveness, quality of delivery* and *integration*, than other sectors (mainly secondary and secondary vocational). This was most prominent for aspects related to engagement of all employees (e.g. onboarding) and alignment with the school vision. Likely explanations can be found in scale differences, with secondary and secondary vocational schools having a larger number of students and staff. Lines of communication among and involvement of employees in primary schools are, therefore, easier to establish (Gleddie, 2011; Adamowitsch, Gugglberger, and Dür, 2014). Teachers also generally spend all day with their students and, therefore, may have more ownership and a comprehensive view of the curriculum, rather than secondary (vocational) schools where the approach is often more fragmented (Adamowitsch, Gugglberger, and Dür, 2014). Similarities herein between primary and special needs schools may explain the lack of differences found between these school types. Altogether, these results point to more research to understand how to improve HPS implementation in secondary and secondary vocational schools.

### Strengths and limitations

The use of a validated questionnaire to assess seven implementation dimensions is a major strength of the present study, as previous research focused only on the dimensions of *adherence* and/or *dose* and often didn’t use a validated measure (Langford et al., 2014). Moreover, the study design and interpretation of results were conducted in close collaboration with practitioners in the CoP, allowing for optimal tailoring to Dutch school health promotion practice (Wenger, 1998). Additionally, the study sample included schools from four school types and three HS categories, providing crucial insights into under-researched school types (Keshavarz Mohammadi, Rowling, and Nutbeam, 2010; Vilaca et al., 2019).

A main limitation concerns data collection among just one respondent per school. Among all respondents, scores may also be higher due to social desirability bias from the self-reported questionnaire. Nonetheless, this was considered the most appropriate sample, because collection among multiple employees was not feasible, often only a few employees were knowledgeable on the topic, and respondents were encouraged to jointly fill out the questionnaire. Secondly, the overall response rate among invited schools was lower compared to similar studies (Rozema et al., 2018; Lassen et al., 2019; Dadaczynski et al., 2020), probably due to the second COVID wave in the Netherlands during data collection. Thirdly, the time of lockdown was not considered representative of the degree of implementation, and it is unclear if and how the lockdown may have affected the degree of implementation. Fourthly, results may have been affected by missing data, although this is expected to be limited as it was roughly evenly divided over subgroups. Fifthly, the results may be less generalizable because of the limited representativeness of the sample. The percentage of schools that ever obtained an HS Program certificate (43%) is higher than the national percentage in 2019 (19%) (Programma Gezonde School, 2020), which likely led to higher average scores for the total sample as well as school types. As compared to all schools in the seven regions, more schools in a low urbanicity area (46% relative to 26%) and fewer in high urbanicity (37% relative to 63%) were recruited, possibly resulting in higher average *adherence* to environment items (e.g. due to more sports facilities and fewer food outlets; van Dongen et al., 2021). Additionally, the sample includes more Catholic (43% relative to 27%) and fewer public schools (19% relative to 29%) possibly leading to differences in pedagogical vision. Finally, as this is the first application of the HPS implementation questionnaire, future studies are needed to confirm the robustness of the results.

## CONCLUSION

The degree of implementation of whole-school health promotion showed large variation and much room for improvement in the majority of schools. Schools are working the most on aspects that are closely linked to the pedagogical vision. Less is achieved for aspects related to broader implementation, including adherence and involvement of all actors. There were modest yet consistent differences between schools that do and don’t identify as (certified) HPS, mainly in adherence and coordination. There is still room for improvement in a broader perspective on health and more insight into (other) conditions for effectiveness. Finally, more research is needed to improve implementation in secondary and secondary vocational schools.

## Supplementary Material

daad173_suppl_Supplementary_Files_1

daad173_suppl_Supplementary_Files_2

daad173_suppl_Supplementary_Files_3

daad173_suppl_Supplementary_Files_4
