# Supplementary material for: Fidelity, adaptation and integration of whole-school health promotion within Dutch schools: a cross-sectional survey study
Source: Health Promot Int. 2023 Dec 20;38(6):daad173. doi: 10.1093/heapro/daad173 (PMC10733658; doi:10.1093/heapro/daad173)
Supplement: daad173_suppl_Supplementary_Files_1 [file daad173_suppl_supplementary_files_1.docx]

# Supplementary file 1: Questionnaire as distributed

## Overview of the HPS Implementation Questionnaire

| Dimension | Definition | Number of items | Example question |
| --- | --- | --- | --- |
| Adherence | The extent to which program components were delivered according to underlying theoretical guidelines. | 13 (of which 7 per topic) | For which of the following topics did the layout of the school  environment improve the health and wellbeing of pupils/students? |
| Dose | The amount of program content received by the target group. | 7 | Members of staff at my school or school location clearly set a good example to pupils/students in the area of health and wellbeing. |
| Participant responsiveness | The degree to which the target group is engaged with the program. | 9 | There was active participation in the 'school approach towards health and wellbeing' by.... |
| Quality of delivery | How the program is delivered. | 5 | Teachers in my school or school location were on average competent enough to implement the 'school approach towards health and wellbeing'. |
| Program differentiation | The extent to which the program contains unique elements to achieve effective outcomes. | 1 | There were specific activities or unique components in the 'school approach towards health and wellbeing' in my school or school location that, in my opinion, clearly contributed to the health and wellbeing of pupils/students. |
| Adaptation | The extent to which adaptations were made to the program. | 1 | My school or school location tailored the 'school approach towards health and wellbeing' to the specific characteristics and circumstances of my school. |
| Integration | The extent to which the approach is part of school routines, norms, and identity. | 3 | Within my school or school location, paying attention to the health and wellbeing of pupils/students was self-evident. |
| *Degree of implementation* | *Weighted average of the seven dimensions.* |  |  |

*Based on Vennegoor et al. (2022)¹.*

## Introduction

*Dear Madam, Sir,*

*Thank you for taking the time to fill out this questionnaire.*

***What is the purpose?***

*With this survey we will gain a better understanding of schools' approach to lifestyle, health and well-being of pupils/students. We include schools which do and do not work according to the so-called Healthy School Approach (i). With this, we can give advice to municipalities and Public Health Services, among others, to improve the support system for schools. The councils for primary, secondary, and secondary vocational education support this research. Originators include the ministries of Health, Welfare and Sport, and Education, Culture and Science.*

***What will my school or school location receive?***

*You can indicate at the end of the questionnaire that you would like to receive a report. In the report, you can see how your school is doing on each of the components, compared to the national average.*

***Who completes it?***

*The person who knows the most about what is done on student health and wellbeing at your school or school location. For instance, this could be a care coordinator, Healthy School coordinator or principal. You can involve colleagues in filling it out to get as complete a picture as possible.*

***Based on which time frame should I complete this?***

*Answer the questions about the situation in the 2019/2020 school year before the Corona crisis, i.e. August 2019 to February 2020.*

***How long will it take to complete?***

*It takes about 10 to 15 minutes to complete. You can also stop in between and continue at a later time (maximum 1 week). The questionnaire consists of 6 sections. You can see how far along you are in the progress bar at the top of the page.*

***Are my answers anonymous?***

*Your answers are processed completely anonymously. Only the researchers can link your answers to the name of your school or school location. If used for publications, they can never be traced back to you or your school or school location. Your answers will be stored in a secure environment for up to 15 years after completion of the study. Participation is completely voluntary. You can quit the questionnaire at any time without consequences.*

***Questions?***

*Do you have any questions about the study? If so, please contact the researcher, Gerjanne Vennegoor, at g.vennegoor@maastrichtuniversity.nl or XXX XXXXXXX.*

*I agree to participate in this study and the processing of my data*

*□ Yes*

*□ No*

*i=The Healthy School Approach was developed as part of the national Healthy School program. The approach helps schools to work structurally on a healthy lifestyle of pupils/students in order to increase their health and well-being. Schools can be supported in this by a Healthy School adviser from the Public Health Service, or get started themselves with the information and materials on the website.*

## Background questions

1. What is the name of your school?

*Please note! If you work at multiple locations, please complete a separate questionnaire for each location.*

*This is because locations may differ in their answers. You can do this by going to [website] again after completing the questionnaire. A direct link is also available at the end of the questionnaire.*

□ Open answer

2. What is the name of your school location? (if applicable)

□ Open answer

3. In which municipality is your school or school location located?

□ (choose from a list of all municipalities in the Netherlands)

4. Which educational sector is your school or school location part of? (multiple answers possible)

□ Primary school

□ Special school for primary education

□ School for special education

□ Secondary school

□ Special school for secondary education

□ Secondary vocational school

5. What is your position within your school or school location? (multiple answers possible)

□ Board

□ Principal

□ Deputy director

□ Team manager

□ Teacher/Lecturer

□ Physical activity teacher (primary schools)

□ School counselor

□ Care coordinator

□ Healthy School coordinator

□ Facility service

□ Teaching assistant

□ Policy adviser/officer

□ Internship coordinator

□ Support staff

□ Other

6. Are you one of the people who knows the most about the 'school approach towards health and wellbeing' at your school or school location?

*i= By ‘school approach towards health and wellbeing’ we mean everything a school or school location does to promote the lifestyle, health, and wellbeing of pupils/students. This includes at least, but not exclusively, the topics of nutrition, physical activity, wellbeing, relationships and sexuality, smoking, alcohol- and drug prevention, environment, prevention of hearing damage, and media literacy. It does not concern the legal obligations for the curriculum or other legal obligations.*

□ Yes

□ No


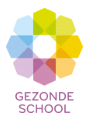


7. Does your school or school location (partially) work according to the principles of the Healthy School Approach?

*i=The Healthy School Approach was developed as part of the national Healthy School program. The approach helps schools to work*

*structurally on a healthy lifestyle of pupils/students in order to increase their health and well-being. Schools can be supported in this by*

*a Healthy School adviser from the Public Health Service, or get started themselves with the information and materials on the website.*

□ Yes

□ No

□ Don’t know

## Adherence to HPS principles

|  | Nutrition | Physical activity | Wellbeing | Smoking, alcohol- and drugs | Relations and sexuality | Prevention of hearing damage | Environment | Media literacy | None | Don’t know |
| --- | --- | --- | --- | --- | --- | --- | --- | --- | --- | --- |
| 8. For which of the following topics was a periodic measurement performed under pupils/students (for example, every year or every two years)?  *For example via a ‘school health profile’, if necessary conducted by the municipal health authorities or similar institute .* | □ | □ | □ | □ | □ | □ | □ | □ | □ | □ |
| 9. For which of the following topics did the layout of the school environment improve the health and wellbeing of pupils/students?  *For example, a healthy selection in the canteen, a ‘green’ playground or a safer school climate.* | □ | □ | □ | □ | □ | □ | □ | □ | □ | □ |
| [new screen] | | | | | | | | | | |
| 10. For which of the following topics were existing teaching methods used in the lessons (for example one or more teaching methods per year in each class)?  *For example, to expand the knowledge of pupils/students, develop skills or to encourage a positive attitude with respect to the topic.* | □ | □ | □ | □ | □ | □ | □ | □ | □ | □ |
| 11. For which of the following topics were periodic supplementary educational activities performed, such as a project week, outing or guest speaker (for example, one or more activities per year in each class)?  *For example, to expand the knowledge of pupils/students, develop skills or to encourage a positive attitude with respect to the topic.* | □ | □ | □ | □ | □ | □ | □ | □ | □ | □ |
| [new screen] | | | | | | | | | | |
| 12. For which of the following topics were members of staff at your school or school location able to prematurely identify any problems that individual pupils/students may have had and to make these open for discussion? | □ | □ | □ | □ | □ | □ | □ | □ | □ | □ |
| 13. For which of the following topics were rules of conduct for members of staff, pupils/students and/or parents/carers present?  *For example regarding use of telephones, serving alcoholic drinks during certificate awards ceremonies, lunchbreaks, birthday treats or interactions with each other.* | □ | □ | □ | □ | □ | □ | □ | □ | □ | □ |
| 14. Which of the topics were a part of school policy as stated in, for example, the school directory, the school plan or the website? | □ | □ | □ | □ | □ | □ | □ | □ | □ | □ |

[new screen]

|  | Board | Management | Workgroup/coordinator | Teachers/lecturers | Support staff | Pupils/students | Parents/carers | External partners | External adviser | None | Don’t know |
| --- | --- | --- | --- | --- | --- | --- | --- | --- | --- | --- | --- |
| 15. With which parties is the 'school approach towards health and wellbeing' evaluated at least annually at your school or school location? (multiple responses possible)  *For example by conducting satisfaction surveys or verbal assessment moments.*  *By ‘school approach towards health and wellbeing’ we mean everything a school or school location does to promote the lifestyle, health, and wellbeing of pupils/students. This includes at least, but not exclusively, the topics of nutrition, physical activity, wellbeing, relationships and sexuality, smoking, alcohol- and drug prevention, environment, prevention of hearing damage, and media literacy.* *It does not concern the legal obligations for the curriculum or other legal obligations.* | □ | □ | □ | □ | □ | □ | □ | □ | □ | □ | □ |

[new screen]

Indicate how much you agree with the statements below.

|  | 1 Definitely not | 2 | 3 | 4 | 5 Definitely | N/A / Don’t know |
| --- | --- | --- | --- | --- | --- | --- |
| 16. In the past academic year, areas of concern arising from the evaluation(s) of the 'school approach towards health and wellbeing' were actively addressed. | □ | □ | □ | □ | □ | □ |
| [new screen] | | | | | | |
| 17. One or more persons actively coordinated the 'school approach towards health and wellbeing' at my school or school location. | □ | □ | □ | □ | □ | □ |
| 18. The responsible person or persons were given enough hours to work on the 'school approach towards health and wellbeing'. | □ | □ | □ | □ | □ | □ |
| 19. There were sufficient funds available for the 'school approach towards health and wellbeing'. | □ | □ | □ | □ | □ | □ |

## Dose

Indicate how much you agree with the statements below.

|  | 1 Definitely not | 2 | 3 | 4 | 5 Definitely |
| --- | --- | --- | --- | --- | --- |
| 20. (Almost) all pupils/students in my school or school location were reached with the 'school approach towards health and wellbeing'. | □ | □ | □ | □ | □ |
| 21. Members of staff at my school or school location clearly set a good example to pupils/students in the area of health and wellbeing.  *For example by eating healthily, not smoking in front of pupils/students, or creating a safe school climate.* | □ | □ | □ | □ | □ |
| 22. Members of staff at my school or school location adequately complied with the rules of conduct and the school policy regarding the health and wellbeing of pupils/students.  *For example by holding pupils/students, parents/carers and/or colleagues to account.* | □ | □ | □ | □ | □ |
| 23. The 'school approach towards health and wellbeing' was placed regularly on the agenda in meetings with members of staff at my school or school location.  *For example, several times a year.* | □ | □ | □ | □ | □ |
| [new screen] | | | | | |
| 24. Within my school or school location there was active communication about the 'school approach towards health and wellbeing' with ...  *For example, several times a year. For example via the website, email or meetings.*  … staff  … pupils/students  … parents/carers |  | | | | |
|  |  | | | | |
|  | □ | □ | □ | □ | □ |
|  | □ | □ | □ | □ | □ |
|  | □ | □ | □ | □ | □ |

## Participant responsiveness

Indicate how much you agree with the statements below.

|  | 1 Definitely not | 2 | 3 | 4 | 5 Definitely | N/A / Don’t know |
| --- | --- | --- | --- | --- | --- | --- |
| 25. There was active participation in the 'school approach towards health and wellbeing' by....  *For example in workgroups, consultations or requests for advice.* |  | | | | | |
| … board | □ | □ | □ | □ | □ | □ |
| … management | □ | □ | □ | □ | □ | □ |
| … workgroup/coordinator | □ | □ | □ | □ | □ | □ |
| … teachers/lecturers | □ | □ | □ | □ | □ | □ |
| … support staff | □ | □ | □ | □ | □ | □ |
| … pupils/students | □ | □ | □ | □ | □ | □ |
| … parents/carers | □ | □ | □ | □ | □ | □ |
| … external partners | □ | □ | □ | □ | □ | □ |
| … external adviser | □ | □ | □ | □ | □ | □ |

## Quality of delivery

Indicate how much you agree with the statements below.

|  | 1 Definitely not | 2 | 3 | 4 | 5 Definitely | N/A / Don’t know |
| --- | --- | --- | --- | --- | --- | --- |
| 26. Teachers in my school or school location were on average competent enough to implement the 'school approach towards health and wellbeing'.  *By competence we mean the knowledge and experience that teachers have.* | □ | □ | □ | □ | □ | □ |
| 27. On average, external professionals had sufficient expertise to implement the 'school approach towards health and wellbeing'.  *By expertise we mean the knowledge and experience that external professionals possess.* | □ | □ | □ | □ | □ | □ |
| 28. New staff members at my school or school location were informed about the 'school approach towards health and wellbeing'. | □ | □ | □ | □ | □ | □ |
| 29. There was regular contact between my school or school location and external supporters about the 'school approach towards health and wellbeing' (for example several times a year).  *For example, by telephone, email or during appointments. External supporters are, for example, a Healthy School adviser, a community sports coach or a welfare worker.* | □ | □ | □ | □ | □ | □ |
| 30. There was sufficient ownership of the 'school approach towards health and wellbeing' among members of staff in my school or school location.  *Ownership means taking responsibility for the process and making a clear contribution to it.* | □ | □ | □ | □ | □ | □ |

## Integration, program differentiation, adaptation

Indicate how much you agree with the statements below.

|  | 1 Definitely not | 2 | 3 | 4 | 5 Definitely |
| --- | --- | --- | --- | --- | --- |
| 31. The core values, vision, or mission of my school or school location were in line with working toward health and wellbeing of the pupils/students.  *The core values, vision and/or mission of a school or school location are usually listed on the website and/or in the school plan.* | □ | □ | □ | □ | □ |
| 32. Pupils/students at my school or school location were facilitated in making healthy choices concerning health and wellbeing.  *For example, by not (or rarely) offering unhealthy options or because the (social or physical) school environment is arranged in such a way that healthy choices are made as a matter of course.* | □ | □ | □ | □ | □ |
| 33. Within my school or school location, paying attention to the health and wellbeing of pupils/students was self-evident.  *For example when organizing an activity or a change in the (physical or social) school environment.* | □ | □ | □ | □ | □ |
| 34. There were specific activities or unique components in the 'school approach towards health and wellbeing' in my school or school location that, in my opinion, clearly contributed to the health and wellbeing of pupils/students.  *For example, the organization of an educational activity around health and wellbeing or the design of the (physical or social) school environment* | □ | □ | □ | □ | □ |
| 35. My school or school location tailored the 'school approach towards health and wellbeing' to the specific characteristics and circumstances of my school.  *For example, the needs or background of pupils/students and teachers/lecturers, the organizational structure of the school or the possibilities provided by the school building.* | □ | □ | □ | □ | □ |

## Scoring scheme

| **Adherence score (0-4)**  *(((4/56) * Sum topic scores) + ((Score evaluation + Sum items 9 to 12) / 5)) / 2* |  |
| --- | --- |
| Sum topic scores (0-56)  *# of times the topics were selected in Q8-14* |  |
| Score evaluation (0-4)  *(4 / (9 - # of parties N/A in Q25)) * # of parties selected* |  |
| Sum points for items 16 to 19 (0-16)  *1 Definitely not = 0 points; 2 = 1 point; 3 = 2 points; 4 = 3 points; 5 Definitely = 4 points* |  |
| **Dose score (0-4)**  *(Sum points for items 20 to 24) / 7*  *1 Definitely not = 0 points; 2 = 1 point; 3 = 2 points; 4 = 3 points; 5 Definitely = 4 points* |  |
| **Participant responsiveness score (0-4)**  *(Sum points for item 25) / (9 - # of parties N/A)*  *1 Definitely not = 0 points; 2 = 1 point; 3 = 2 points; 4 = 3 points; 5 Definitely = 4 points* |  |
| **Quality of delivery score (0-4)**  (Sum points for items 26 to 30) / (5 - # of items N/A)  *1 Definitely not = 0 points; 2 = 1 point; 3 = 2 points; 4 = 3 points; 5 Definitely = 4 points* |  |
| **Integration score (0-4)**  (Sum points for items 31 to 33) / 3  *1 Definitely not = 0 points; 2 = 1 point; 3 = 2 points; 4 = 3 points; 5 Definitely = 4 points* |  |
| **Program differentiation score (0-4)**  Points for item 34  *1 Definitely not = 0 points; 2 = 1 point; 3 = 2 points; 4 = 3 points; 5 Definitely = 4 points* |  |
| **Adaptation score (0-4)**  Points for item 28  *1 Definitely not = 0 points; 2 = 1 point; 3 = 2 points; 4 = 3 points; 5 Definitely = 4 points* |  |
| **Degree of implementation (0-4)**  (Adherence score + Dose score + Participant responsiveness score + Quality of delivery score + Integration score + (0.5 * Program differentiation score) + (0.5 * Adaptation score)) / 6 |  |
