# Supplementary material for: Fidelity, adaptation and integration of whole-school health promotion within Dutch schools: a cross-sectional survey study
Source: Health Promot Int. 2023 Dec 20;38(6):daad173. doi: 10.1093/heapro/daad173 (PMC10733658; doi:10.1093/heapro/daad173)
Supplement: daad173_suppl_Supplementary_Files_2 [file daad173_suppl_supplementary_files_2.docx]

**Supplementary file 2 – Characteristics of the study sample**

**Table 1. Characteristics of the study sample**

|  | **N** | **Primary schools (N=365)** | **Secondary schools (N=102)** | **Secondary vocational schools (N=25)** | **Special needs schools (N=43)** | **Total (N=535)** |
| --- | --- | --- | --- | --- | --- | --- |
|  |  | ***% / M (±SD)*** | ***% / M (±SD)*** | ***% / M (±SD)*** | ***% / M (±SD)*** | ***% / M (±SD)*** |
| **Responses**  Response percentage  Complete responses | 535 |  |  |  |  |  |
|  | % | 21.5 | 28.2 | 13.2 | 27.9 | 22.3 |
|  |  | 78.9 | 73.5 | 88.0 | 76.7 | 78.1 |
| **HS category**  No HS  Partial HS | 535 |  |  |  |  |  |
|  | %  163 | 37.3 | 15.7 | 28.0 | 9.3 | 30.5 |
|  | 144 | 24.9 | 33.3 | 20.0 | 32.6 | 26.9 |
| Certified HS | 228 | 37.8 | 51.0 | 52.0 | 58.1 | 42.6 |
| **Professional role** | 535 |  |  |  |  |  |
| Principal | % | 51.2 | 12.7 | 4.0 | 11.6 | 38.5 |
| School counselor |  | 16.7 | 1.0 | 4.0 | 11.6 | 12.7 |
| Teacher/Lecturer |  | 14.2 | 32.4 | 28.0 | 20.9 | 18.9 |
| ‘Healthy School’ coordinator |  | 12.9 | 29.4 | 36.0 | 34.9 | 18.9 |
| Deputy director |  | 8.5 | 6.9 | 0.0 | 2.3 | 7.3 |
| Other |  | 6.3 | 8.8 | 16.0 | 4.7 | 7.1 |
| Team manager |  | 5.2 | 12.7 | 28.0 | 20.9 | 9.0 |
| Physical education teacher |  | 3.8 | 3.9 | 0.0 | 18.6 | 4.9 |
| Internship coordinator |  | 2.2 | 0 | 4.0 | 4.7 | 2.1 |
| Board |  | 1.6 | 0.0 | 0.0 | 0.0 | 1.1 |
| Care coordinator |  | 1.1 | 23.5 | 4.0 | 4.7 | 5.8 |
| Support staff |  | 0.8 | 2.9 | 4.0 | 0.0 | 1.3 |
| Policy adviser/officer |  | 0.6 | 0 | 20.0 | 0.0 | 1.3 |
| Teaching assistant |  | 0.3 | 2.0 | 0.0 | 11.6 | 1.5 |
| Facility service |  | 0.0 | 2.0 | 4.0 | 0.0 | 0.6 |
| **Number of students in schools** | 501  M (±SD) | 233.6 (±137.2) | 828.0 (±542.8) | - | 153.1 (±79.8) | 346.6 (±361.6) |
| **Mean percentage of students with two lower educated parents** | 337  M (±SD) | 9.2 (±10.6) | - | - | - | - |
| **School type** | 506 |  |  |  |  |  |
| Catholic | % | 45.0 | 30.7 | N/A | 51.2 | 42.5 |
| Protestant |  | 27.7 | 20.8 |  | 9.3 | 24.5 |
| Public |  | 20.4 | 19.8 |  | 7.0 | 19.0 |
| Independent non-denominational |  | 5.3 | 11.9 |  | 27.9 | 8.5 |
| Collaboration |  | 0.6 | 15.8 |  | 0.0 | 4.2 |
| Other |  | 1.1 | 1.0 |  | 4.7 | 1.4 |
| **Level of urbanicity** | 523 |  |  |  |  |  |
| Low | % | 41.1 | 55.4 | 66.7 | 53.5 | 45.9 |
| Moderate |  | 14.8 | 22.8 | 28.6 | 20.9 | 17.4 |
| High |  | 44.1 | 21.8 | 4.8 | 25.6 | 36.7 |

*For secondary vocational schools, data on number of students are not available. Data on percentage of lower educated parents are only available for primary schools. Level of urbanicity: low (<1000 addresses per km²), moderate (1000 to 1500), high (>1500). N/A = not applicable, M = mean, SD = standard deviation.*
