# Supplementary material for: Fidelity, adaptation and integration of whole-school health promotion within Dutch schools: a cross-sectional survey study
Source: Health Promot Int. 2023 Dec 20;38(6):daad173. doi: 10.1093/heapro/daad173 (PMC10733658; doi:10.1093/heapro/daad173)
Supplement: daad173_suppl_Supplementary_Files_3 [file daad173_suppl_supplementary_files_3.docx]

**Supplementary file 3 – Item scores**

**Table 2. (Sub)group implementation statistics per item and (sub)scale**

|  | **School type** | | | | | | | | **HS category** | | | | | | **Total** | |
| --- | --- | --- | --- | --- | --- | --- | --- | --- | --- | --- | --- | --- | --- | --- | --- | --- |
| **Item ͣ** | **Primary schools** | | **Secondary schools** | | **Secondary vocational schools** | | **Special needs schools** | | **No HS** | | **Partial HS** | | **Certified HS** | |  |  |
|  | **N** | **M (±SD)** | **N** | **M (±SD)** | **N** | **M (±SD)** | **N** | **M (±SD)** | **N** | **M (±SD)** | **N** | **M (±SD)** | **N** | **M (±SD)** | **N** | **M (±SD)** |
| **Adherence** |  |  |  |  |  |  |  |  |  |  |  |  |  |  |  |  |
| Topic score Nutrition | 311 | 1.92 (±1.12) | 80 | 1.97 (±1.17) | 22 | 1.79 (±1.30) | 35 | 1.78 (±1.10) | 142 | 1.38 (±1.00) | 108 | 1.97 (±1.04) | 198 | 2.26 (±1.13) | 448 | 1.91 (±1.13) |
| Topic score Physical activity | 311 | 2.11 (±1.16) | 80 | 1.68 (±1.06) | 22 | 1.61 (±1.25) | 35 | 1.96 (±1.09) | 142 | 1.66 (±1.04) | 108 | 1.94 (±1.04) | 198 | 2.28 (±1.21) | 448 | 2.00 (±1.15) |
| Topic score Wellbeing | 311 | 2.94 (±0.89) | 80 | 2.14 (±1.13) | 22 | 2.08 (±1.11) | 35 | 2.37 (±1.17) | 142 | 2.74 (±0.98) | 108 | 2.64 (±0.99) | 198 | 2.73 (±1.09) | 448 | 2.71 (±1.03) |
| Topic score Smoking, alcohol & drugs | 311 | 0.67 (±0.81) | 80 | 2.44 (±1.01) | 22 | 2.65 (±1.01) | 35 | 1.31 (±1.24) | 142 | 0.88 (±0.99) | 108 | 1.32 (±1.24) | 198 | 1.22 (±1.23) | 448 | 1.13 (±1.17) |
| Topic score Relations & sexuality | 311 | 0.91 (±0.94) | 80 | 1.63 (±1.00) | 22 | 1.09 (±1.28) | 35 | 1.40 (±1.09) | 142 | 0.92 (±0.90) | 108 | 1.17 (±1.07) | 198 | 1.16 (±1.07) | 448 | 1.09 (±1.02) |
| Topic score Prevention of hearing damage | 311 | 1.92 (±1.17) | 80 | 1.71 (±1.02) | 22 | 1.81 (±1.38) | 35 | 1.75 (±1.09) | 142 | 2.00 (±1.09) | 108 | 1.88 (±1.21) | 198 | 1.75 (±1.15) | 448 | 1.86 (±1.15) |
| Topic score Environment | 311 | 0.94 (±1.00) | 80 | 0.62 (±0.79) | 22 | 0.42 (±0.59) | 35 | 0.54 (±0.83) | 142 | 0.89 (±0.94) | 108 | 1.01 (±1.05) | 198 | 0.68 (±0.88) | 448 | 0.83 (±0.95) |
| Topic score Media literacy | 311 | 1.82 (±1.89) | 80 | 2.13 (±1.01) | 22 | 1.32 (±1.13) | 35 | 1.40 (±1.09) | 142 | 1.86 (±1.03) | 108 | 1.87 (±1.15) | 198 | 1.76 (±1.10) | 448 | 1.82 (±1.09) |
| Annual evaluation | 288 | 1.59 (±0.89) | 70 | 1.19 (±0.74) | 16 | 1.39 (±0.60) | 31 | 1.35 (±0.77) | 119 | 1.41 (±0.94) | 100 | 1.50 (±0.83) | 186 | 1.55 (±0.81) | 405 | 1.49 (±0.86) |
| One or more coordinators | 305 | 2.95 (±1.16) | 77 | 2.94 (±1.21) | 21 | 2.76 (±0.89) | 33 | 3.36 (±0.82) | 133 | 2.28 (±1.29) | 106 | 2.96 (±1.03) | 197 | 3.44 (±0.80) | 436 | 2.97 (±1.14) |
| Sufficient hours available | 292 | 2.36 (±1.18) | 69 | 1.93 (±1.32) | 20 | 1.95 (±1.00) | 32 | 2.50 (±1.16) | 124 | 2.07 (±1.22) | 99 | 2.11 (±1.13) | 190 | 2.51 (±1.20) | 413 | 2.28 (±1.21) |
| Sufficient budget available | 285 | 2.33 (±1.16) | 71 | 2.13 (±1.24) | 18 | 2.11 (±1.13) | 31 | 2.81 (±1.05) | 118 | 2.18 (±1.14) | 103 | 2.14 (±1.19) | 184 | 2.51 (±1.16) | 405 | 2.32 (±1.17) |
| Making good use of feedback | 269 | 2.64 (±0.93) | 64 | 2.36 (±1.01) | 16 | 2.38 (±0.96) | 28 | 2.39 (±0.83) | 104 | 2.34 (±0.99) | 93 | 2.47 (±0.94) | 180 | 2.73 (±0.88) | 377 | 2.56 (±0.94) |
| *Score adherence* | *298* | *2.01 (±0.60)* | *76* | *1.98 (±0.53)* | *22* | *1.86 (±0.51)* | *33* | *2.05 (±0.53)* | *130* | *1.78 (±0.64)* | *105* | *1.99 (±0.51)* | *194* | *2.16 (±0.53)* | *429* | *2.00 (±0.58)* |
| **Dose** |  |  |  |  |  |  |  |  |  |  |  |  |  |  |  |  |
| Reach of (almost) all students | 303 | 2.95 (±0.97) | 80 | 2.25 (±1.15) | 22 | 2.00 (±0.87) | 34 | 2.59 (±0.93) | 134 | 2.49 (±1.15) | 106 | 2.63 (±1.08) | 199 | 2.98 (±0.89) | 439 | 2.74 (±1.04) |
| Employees give a good example | 303 | 3.11 (±0.77) | 80 | 2.39 (±0.91) | 22 | 2.18 (±0.50) | 34 | 2.68 (±0.88) | 134 | 2.92 (±0.93) | 106 | 2.82 (±0.88) | 199 | 2.92 (±0.79) | 439 | 2.90 (±0.86) |
| Employees adhere to code of conduct | 303 | 3.23 (±0.70) | 80 | 2.64 (±0.73) | 22 | 2.32 (±0.72) | 34 | 2.76 (±0.65) | 134 | 3.01 (±0.89) | 106 | 3.06 (±0.73) | 199 | 3.05 (±0.68) | 439 | 3.04 (±0.76) |
| Regular agenda item for employees | 303 | 2.35 (±1.10) | 80 | 1.71 (±1.08) | 22 | 1.86 (±0.94) | 34 | 2.26 (±1.16) | 134 | 1.88 (±1.18) | 106 | 2.14 (±1.01) | 199 | 2.45 (±1.08) | 439 | 2.20 (±1.12) |
| Active communication to students | 303 | 2.85 (±0.96) | 80 | 2.13 (±1.14) | 22 | 2.14 (±0.77) | 34 | 2.71 (±0.84) | 134 | 2.46 (±1.07) | 105 | 2.60 (±0.96) | 199 | 2.85 (±0.99) | 438 | 2.67 (±1.02) |
| Active communication to parents | 302 | 2.79 (±0.90) | 80 | 1.28 (±1.04) | 22 | 1.95 (±0.65) | 34 | 2.50 (±0.75) | 134 | 2.39 (±1.00) | 105 | 2.67 (±0.83) | 199 | 2.77 (±0.92) | 438 | 2.63 (±0.94) |
| Active communication to employees | 302 | 2.61 (±0.87) | 80 | 1.98 (±0.91) | 22 | 1.36 (±1.09) | 34 | 2.06 (±0.85) | 134 | 2.22 (±0.96) | 105 | 2.31 (±0.88) | 199 | 2.54 (±0.97) | 438 | 2.39 (±0.96) |
| *Score dose* | *302* | *2.84 (±0.69)* | *80* | *2.19 (±0.75)* | *22* | *1.97 (±0.49)* | *34* | *2.51 (±0.61)* | *134* | *2.48 (±0.82)* | *105* | *2.60 (±0.67)* | *199* | *2.80 (±0.71)* | *438* | *2.65 (±0.75)* |
| **Participant responsiveness** |  |  |  |  |  |  |  |  |  |  |  |  |  |  |  |  |
| Active involvement of management | 262 | 1.90 (±1.22) | 64 | 1.50 (±1.17) | 22 | 2.14 (±0.94) | 27 | 1.59 (±1.22) | 117 | 1.91 (±1.28) | 93 | 1.85 (±1.21) | 165 | 1.75 (±1.16) | 375 | 1.83 (±1.21) |
| Active involvement of school principal | 298 | 3.27 (±0.86) | 76 | 2.57 (±1.12) | 22 | 2.64 (±0.90) | 34 | 2.59 (±0.99) | 131 | 2.86 (±1.01) | 105 | 3.06 (±0.94) | 194 | 3.20 (±0.96) | 430 | 3.06 (±0.98) |
| Active involvement of coordinator | 262 | 3.16 (±1.12) | 72 | 2.97 (±1.09) | 20 | 2.85 (±1.04) | 31 | 3.32 (±0.79) | 105 | 2.56 (±1.32) | 98 | 3.05 (±1.05) | 182 | 3.48 (±0.78) | 385 | 3.12 (±1.09) |
| Active involvement of teachers | 299 | 2.96 (±0.86) | 76 | 2.55 (±0.90) | 22 | 2.23 (±0.92) | 34 | 2.76 (±0.65) | 132 | 2.67 (±0.95) | 105 | 2.83 (±0.88) | 194 | 2.95 (±0.80) | 431 | 2.84 (±0.88) |
| Active involvement of support staff | 285 | 2.49 (±1.10) | 76 | 2.33 (±1.11) | 22 | 2.14 (±0.94) | 34 | 2.76 (±0.92) | 127 | 2.28 (±1.18) | 100 | 2.40 (±1.06) | 190 | 2.62 (±1.02) | 417 | 2.46 (±1.09) |
| Active involvement of students | 299 | 2.40 (±1.01) | 76 | 2.17 (±0.96) | 22 | 2.09 (±0.61) | 34 | 2.12 (±0.91) | 133 | 2.15 (±1.08) | 104 | 2.35 (±1.02) | 194 | 2.43 (±0.88) | 431 | 2.32 (±0.98) |
| Active involvement of parents | 298 | 2.20 (±0.94) | 77 | 1.79 (±0.91) | 21 | 1.00 (±0.71) | 34 | 1.50 (±0.83) | 132 | 1.89 (±0.95) | 105 | 1.99 (±1.00) | 193 | 2.11 (±0.95) | 430 | 2.01 (±0.97) |
| Active involvement of external partners | 268 | 2.22 (±1.22) | 72 | 2.11 (±1.13) | 20 | 2.15 (±1.14) | 30 | 1.80 (±1.24) | 117 | 1.81 (±1.21) | 99 | 2.04 (±1.12) | 174 | 2.47 (±1.17) | 390 | 2.16 (±1.20) |
| Active involvement of external adviser | 250 | 2.00 (±1.35) | 71 | 2.24 (±1.30) | 18 | 2.22 (±1.00) | 30 | 1.93 (±1.20) | 106 | 1.42 (±1.29) | 94 | 1.94 (±1.23) | 169 | 2.51 (±1.20) | 369 | 2.05 (±1.32) |
| *Score participant responsiveness* | *300* | *2.53 (±0.72)* | *77* | *2.27 (±0.68)* | *22* | *2.16 (±0.53)* | *34* | *2.27 (±0.59)* | *133* | *2.21 (±0.80)* | *105* | *2.41 (±0.66)* | *195* | *2.62 (±0.62)* | *433* | *2.44 (±0.71)* |
| **Quality of delivery** |  |  |  |  |  |  |  |  |  |  |  |  |  |  |  |  |
| Expertise of teachers | 291 | 3.02 (±0.75) | 72 | 2.67 (±0.79) | 22 | 2.45 (±0.86) | 33 | 2.85 (±0.80) | 120 | 2.84 (±0.85) | 104 | 2.81 (±0.73) | 194 | 3.02 (±0.76) | 418 | 2.91 (±0.78) |
| Expertise of external professionals | 217 | 3.17 (±0.76) | 62 | 3.13 (±0.71) | 19 | 3.00 (±0.82) | 24 | 3.00 (±0.72) | 86 | 2.91 (±0.79) | 77 | 3.04 (±0.77) | 159 | 3.31 (±0.68) | 322 | 3.14 (±0.75) |
| Notifying new employees | 278 | 2.63 (±1.01) | 67 | 1.61 (±0.83) | 20 | 1.45 (±0.83) | 32 | 2.19 (±0.97) | 109 | 2.33 (±1.04) | 98 | 2.31 (±1.02) | 190 | 2.41 (±1.10) | 397 | 2.36 (±1.06) |
| Regular contact with external supporter(s) | 238 | 2.26 (±1.18) | 64 | 2.55 (±1.17) | 19 | 2.21 (±0.86) | 30 | 2.23 (±1.14) | 90 | 1.80 (±1.18) | 85 | 2.19 (±1.13) | 176 | 2.63 (±1.06) | 351 | 2.31 (±1.16) |
| Sufficient ownership present | 288 | 2.80 (±0.98) | 70 | 2.16 (±0.97) | 22 | 2.00 (±1.02) | 33 | 2.24 (±1.03) | 117 | 2.55 (±1.09) | 102 | 2.56 (±1.02) | 194 | 2.66 (±1.00) | 413 | 2.61 (±1.03) |
| *Score quality of delivery* | *291* | *2.77 (±0.72)* | *72* | *2.41 (±0.64)* | *22* | *2.18 (±0.63)* | *33* | *2.48 (±0.68)* | *120* | *2.53 (±0.78)* | *104* | *2.60 (±0.68)* | *194* | *2.77 (±0.68)* | *418* | *2.66 (±0.72)* |
| **Program differentiation** |  |  |  |  |  |  |  |  |  |  |  |  |  |  |  |  |
| Unique elements | 288 | 2.66 (±1.02) | 75 | 2.63 (±1.08) | 22 | 2.18 (±0.80) | 33 | 2.58 (±0.79) | 123 | 2.33 (±1.09) | 101 | 2.65 (±0.88) | 194 | 2.79 (±0.97) | 418 | 2.62 (±1.00) |
| *Score program differentiation* | *288* | *2.66 (±1.02)* | *75* | *2.63 (±1.08)* | *22* | *2.18 (±0.80)* | *33* | *2.58 (±0.79)* | *123* | *2.33 (±1.09)* | *101* | *2.65 (±0.88)* | *194* | *2.79 (±0.97)* | *418* | *2.62 (±1.00)* |
| **Adaptation** |  |  |  |  |  |  |  |  |  |  |  |  |  |  |  |  |
| Adaptation to school context | 288 | 2.88 (±0.92) | 75 | 2.69 (±0.99) | 22 | 2.41 (±0.91) | 33 | 2.79 (±0.86) | 123 | 2.59 (±1.09) | 101 | 2.78 (±0.78) | 194 | 2.97 (±0.86) | 418 | 2.82 (±0.93) |
| *Score adaptation* | *288* | *2.88 (±0.92)* | *75* | *2.69 (±0.99)* | *22* | *2.41 (±0.91)* | *33* | *2.79 (±0.86)* | *123* | *2.59 (±1.09)* | *101* | *2.78 (±0.78)* | *194* | *2.97 (±0.86)* | *418* | *2.82 (±0.93)* |
| **Integration** |  |  |  |  |  |  |  |  |  |  |  |  |  |  |  |  |
| In line with school vision | 288 | 3.30 (±0.77) | 75 | 3.13 (±0.74) | 22 | 2.55 (±1.06) | 33 | 3.03 (±0.77) | 123 | 3.11 (±0.79) | 101 | 3.20 (±0.84) | 194 | 3.27 (±0.78) | 418 | 3.21 (±0.80) |
| Healthy choices are made easy | 288 | 2.82 (±0.77) | 75 | 2.56 (±0.83) | 22 | 2.36 (±0.79) | 33 | 2.36 (±0.70) | 123 | 2.62 (±0.81) | 101 | 2.64 (±0.77) | 194 | 2.81 (±0.83) | 418 | 2.72 (±0.79) |
| Self-evident character | 288 | 3.00 (±0.83) | 75 | 2.80 (±0.90) | 22 | 2.55 (±0.96) | 33 | 2.73 (±0.88) | 123 | 2.73 (±0.92) | 101 | 2.93 (±0.82) | 194 | 3.03 (±0.97) | 418 | 2.92 (±0.86) |
| *Score integration* | *288* | *3.04 (±0.65)* | *75* | *2.83 (±0.62)* | *22* | *2.48 (±0.73)* | *33* | *2.71 (±0.53)* | *123* | *2.82 (±0.70)* | *101* | *2.92 (±0.60)* | *194* | *3.04 (±0.65)* | *418* | *2.95 (±0.66)* |
| ***Degree of implementation*** |  |  |  |  |  |  |  |  |  |  |  |  |  |  |  |  |
| *Degree of implementation* | *288* | *2.65 (±0.57)* | *75* | *2.36 (±0.57)* | *22* | *2.13 (±0.50)* | *33* | *2.43 (±0.48)* | *123* | *2.36 (±0.66)* | *101* | *2.51 (±0.51)* | *194* | *2.69 (±0.53)* | *418* | *2.55 (±0.58)* |

*Scale 0-4. Statistics in green are the highest average scores within the subgroup (for columns ‘School type’ and ‘HS category’) or subscale (for column ‘Total’); statistics in red the lowest. ͣ Summaries of each item. HS = Healthy School, M = mean, SD = standard deviation*
