# Supplementary material for: Fidelity, adaptation and integration of whole-school health promotion within Dutch schools: a cross-sectional survey study
Source: Health Promot Int. 2023 Dec 20;38(6):daad173. doi: 10.1093/heapro/daad173 (PMC10733658; doi:10.1093/heapro/daad173)
Supplement: daad173_suppl_Supplementary_Files_4 [file daad173_suppl_supplementary_files_4.docx]

**Supplementary file 4 – Detailed adherence scores**

**Table 3. Breakdown of adherence topic-scores per item and topic**

| **Topic** | **Recurrent measurement of student health (%)** | **School environment (%)** | **Use of existing teaching materials (%)** | **Use of educational activities (%)** | **Employees are able to identify problems (%)** | **Rules of conduct available (%)** | **Part of school policy (%)** |
| --- | --- | --- | --- | --- | --- | --- | --- |
| **Nutrition** | 15.5 | 40.2 | 43.6 | 41.7 | 45.0 | 47.1 | 51.4 |
| **Physical activity** | 28.0 | 49.0 | 52.1 | 47.7 | 45.6 | 27.1 | 47.7 |
| **Wellbeing** | 62.8 | 52.7 | 57.2 | 26.9 | 72.5 | 64.7 | 69.5 |
| **Smoking, alcohol & drugs** | 9.2 | 18.9 | 17.9 | 24.1 | 22.1 | 45.2 | 29.5 |
| **Relations & sexuality** | 8.6 | 9.2 | 39.1 | 25.6 | 34.0 | 26.9 | 18.7 |
| **Prevention of hearing damage** | 37.8 | 43.0 | 22.1 | 15.7 | 47.1 | 61.3 | 52.0 |
| **Environment** | 9.7 | 25.4 | 13.1 | 21.5 | 15.0 | 21.9 | 16.4 |
| **Media literacy** | 9.9 | 23.6 | 43.9 | 41.7 | 45.4 | 58.7 | 46.7 |
| **None** | 8.6 | 4.7 | 2.8 | 6.9 | 2.2 | 0.4 | 0.6 |
| **Don't know** | 4.3 | 4.7 | 3.2 | 3.9 | 2.8 | 1.7 | 2.1 |
| ***Total*** | *83.9* | *83.6* | *82.8* | *82.1* | *81.3* | *82.4* | *82.1* |

*N=535 for all 7 items.*

**Table 4. Adherence topic-scores for Certified HS schools with and without corresponding topic certificate**

| **Topic** | **Topic certificate** | | **Other certificate(s)** | |
| --- | --- | --- | --- | --- |
|  | ***N*** | ***M (±SD)*** | ***N*** | ***M (±SD)*** |
| **Nutrition** | 104 | 2.76 (±0.95) | 94 | 1.66 (±1.05) |
| **Physical activity** | 108 | 2.77 (±0.91) | 90 | 1.75 (±1.11) |
| **Wellbeing** | 76 | 3.18 (±0.90) | 122 | 2.61 (±1.03) |
| **Smoking, alcohol, and drugs** | 14 | 3.06 (±1.07) | 184 | 1.07 (±1.12) |
| **Relations & sexuality** | 30 | 2.21 (±1.23) | 168 | 1.00 (±0.96) |
| **Environment** | 2 | - | 196 | - |
| **Prevention of hearing damage** | 5 | - | 193 | - |
| **Media literacy** | - | - | - | - |

*N=198 Certified HS schools. Results are only shown if N in both groups is > 10. There is no topic-certificate for Media literacy. For topics Environment and Prevention of hearing damage, there are only certificates for secondary and secondary vocational schools. HS = Healthy School, M = mean, SD = standard deviation.*
